# A Comparative Analysis of Medical Education Models and Curriculums of A Medical University and A Medical Education Center

**DOI:** 10.31729/jnma.4107

**Published:** 2019-02-28

**Authors:** QU Fanwei, HE Jin, MA Hua, Jiang Yanling, Zhao Wenlan, Virasakdi Chongsuvivatwong, Jiang Runsheng

**Affiliations:** 1 Kunming Medical University, 1168 West Chunrong Road, Yuhua Avenue, Chenggong District, Kunming 650500, Yunnan, P.R.China; 2 Department of Clinical Pharmacy, The First Affiliated Hospital of Kunming Medical University, Kunming, 650032, Yunnan, P.R.China; 3 Faculty of Medicine, Prince of Songkla University, 15 Kanjanavanich Road, Hat Yai, Songkhla 90110 Thailand

**Keywords:** *curriculums*, *Hat Yai*, *Kunming Medical University*, *medical education models*, *Medical Education Center*

## Abstract

**Introduction:**

In order to solve the shortage of competent healthcare manpower at the village level of Yunnan Province, We compared the training mode of Kunming Medical University and The Medical Educational Center, Hat Yai of PSU. The aim of this study is to compare the difference of the two institutions and learn from each other's advantages.

**Methods:**

The review covered relevant policy areas and stipulations governing general practitioner training for both countries. Qualitative research was done by using a questionnaire developed in house by the project team, students from the inaugural cohort at KMU and students from the MECH. In Qualitative research, in-depth interviews were carried out with the teaching administration and students from both schools.

**Results:**

In Kunming Medical University, besides the conventional lectures, teaching methods such as case based learning and problem based learning have been worked into the basic science, laboratory, and clerkship/internship sessions. The desired end product is a general practitioner. The curriculum emphasizes general practice and clinical exposure during the course being guided and informed by the “Undergraduate Medical Education Standard — Clinical Medicine” and the “General Practitioner Training Guidelines” about teaching methods. In Prince of Songkla University, the first and second phases consist of basic science and preclinical integrated topics taught at PSU. For the third and final phase, the students have core clinical modules and selective at MECH where the methods are learner centered, problem based, integrated and set in the context of community primary healthcare practice.

**Conclusions:**

We should start with the integration of the medical disciplines and the humanities, so as to restore the lost “art of doctoring”. We need to integrate the various foundational and clinical disciplines into an organ system based curriculum, not just in form, but also in function and purpose.

## INTRODUCTION

In June 2010, the policy was issued by the China government, which covered two areas of developing a general practitioner: led primary healthcare system, and the fully subsidized medical training of manpower earmarked for service at the rural primary healthcare level.^1^ Medical Educational Center, Hatyai (MECH) of Prince of Songkla University (PSU): Thailand has held its national medical education meeting regularly since 1956, perfect the curriculum, and meet the demand of the situation of the future.^2^ In 1974, the Thai Ministry of Health collaborated with a medical university to implement the “Collaborative Project to Increase Production of Rural Doctors (CPIRD)”.^3^

The proportion of graduated medical students who went back to countryside has risen from 23% in 1994 to 31.5% in 2001.^4^ In order to be familiar with the work environment, the students did their intern in their work place after graduation.

This project increases the proportion of rural origin greatly.^5^

## METHODS

The qualitative research was conducted in two sites Kunming Medical University (KMU) of China and Medical Education Centre (MECH), Hatyei of Prince of Songkla University (PSU) of Thailand which were selected according to similar location service function and economic development level. The survey was carried out from July 23 to August 3 of 2012 in both sides according to the arrangement of the research group. This research has gone through Medical Ethics Committee review of Kunming Medical University. The literature review covered relevant policy areas and stipulations governing general practitioner training both countries. The comparisons between both schools and countries were performed via the reviewing of manpower development plans, curriculum design, teaching plans and methods, student and teaching management policies from both schools.

We investigated 15 staffs of teaching affairs and 100 undergraduates of KMU. We investigated 8 staffs of teaching affairs and 39 undergraduates of PSU.

A qualitative study was conducted in individual deep interview of teaching management staff and general practice students in the two countries. The interview contents include curriculum of general practice, policy carried out, existing problem and improvement advice. Qualitative research also included in-depth interviews which were carried out with teaching administration and students from both schools, exploring the domains of curriculum, policy implementation, existing challenges and recommendations for improvements. A quantitative was conducted in self-administration questionnaire to investigate students in two countries. The questionnaire mainly include: basic family information, desire of college entrance exam, satisfaction of curriculum, willingness of working and so on.

All the data were entered in Microsoft Excel and SPSS17.0 software package was used for statistical analysis. The descriptive statistical analysis was done.

## RESULTS

In program length, the length of undergraduate program in KMU is five year as compared to six year in MECH of PSU (Table 1).

**Table 1.** Comparison of Medical Education System. First stage-Graduation

PSU: 6 - year program			KUM: 5 - year program	
Yr 1-3	3 years	Basic medical sciences and preclinical study	Yr 1-3 first half	2 & half years
			Yr 3 second half -Yr4 first half	one year
Yr 4-5	2 years	Clinical study and practice		
Yr 6	1 year	Clinical Clerkship	Yr 4 second half -Yr5	one & half years
Second stage-- After Graduation				
		PSU	KMU	
Yr 7	Graduated internship under the guidance of senior doctors at provincial hospitals		Yr 6-8	resident standardization training
Yr 8-9	Work as doctor at community hospitals or do residency for specialist			

The Health Ministry of Thailand implemented “the Collaborative Project to Increase Production of Rural Doctors (CPIRD) so as to recruit students from rural areas for rural health service and they were compared with students of normal track (Table 2).

**Table 2.** Curriculum Comparison between Rural-Oriented Medical Students and Normal Track.

KMU	Yr 1-3	Yr 3	Yr 4
	Semester 1-5	Semester 6	Semester 7
Clinical Medical (normal track)		Preclinical Curriculum	Clinical Courses
General Practitioner Curriculum (Rural-Oriented Medical Students)	Same Curriculum	ADD. REQ: Rehabilitation Community preventive health care Community health service management	ADD. REQ: Clinical psychological counseling

**Table:** 

KMU	Yr 4	Yr 5
	Semester 8	Semester 9-10
Clinical Medicine	Clinical Courses	Clinical Clerkship: 1 year (at affiliated hospitals &Teaching hospitals)
Clinical Medicine	Clinical Courses	Clinical Clerkship: 1 year (at affiliated hospitals &Teaching hospitals)
General Practitioner Curriculum	Clinical Clerkship: one year & half (at affiliated hospital &Teaching hospitals, rural hospitals and community health care centers)	
(Rural-Oriented Medical Students)	ADD. REQ: Demonstrate of GP education, strengthen community practice	
	Emergency, Rehabilitation Therapy, Infectious Disease, Stomatology, Psychiatry, Community Practice, Children preventive medical care	

The teaching methods in KMU was teacher-centred and involved conventional lectures as compared to learner-centred means of MECH PSU where use of Problem Based Learning and E-learning is used.

Students on clerkship are also assigned patients to be responsible for and also assigned a faculty mentor on a one-to-one basis or one-to-small group basis. This resulted in opportunities for practice and the formation of professional identity and sense of duty (Table 3).

**Table 3.** Comparison of Clinical Clerkship Schedule.

Subject	PSU (Yr 6) PSU / Hatyai Hospital & Affiliated Hospital	KMU (Yr 4.5, GP) Affiliated hospitals& Teaching hospitals
Internal Medicine	12wk	16wk
Psychiatry		2wk
Surgery	9wk	12wk
Elective	3wk	-
Pediatrics	7wk	8wk
Ob-Gyn	7wk	8wk
Fam Med	3wk	16wk
Emergency	3wk	4wk
Orthopedics	4wk	Included in the surgery
Rehabilitation	-	4wk
Infectious disease	-	2wk
Stomatology	-	2wk
TOTAL	48wk	74wk

The internship in KMU runs from 8^th^ to 10^th^ semester as compared to PSU where it is done during in 6^th^ year (Table 4).

The curriculum design and principles of KMU desires the end product is a general practitioner. The curriculum emphasizes general practice and clinical exposure during the course, being guided and informed by the “Undergraduate Medical Education Standard — Clinical Medicine” and the “General Practitioner Training Guidelines”. In MECH, the emphasis is on developing competency for outpatient consultations at community level. It aims to be generalist and systematic in scope, and consists of core general practice modules, electives, and web based learning.

In KMU, modules are organized according to tracks: they are common core tracks, discipline-specific basic and specialist tracks whereas in MECH it is organized according to organs/systems.

In KMU, domains are assessed at the conclusion of each course- module and rotations include theory and skills assessments. Students must pass a graduation examination integrating all subjects before qualifying for the bachelor's degree. They will be qualified to attempt the China Medical Licensing Examination one year from graduation.

In MECH PSU, the domains of theory and skills are assessed at the conclusion of each module/course. At the conclusion of each phase, the MECH administers summative examinations based on the standards laid out by the Thailand Medical Licensing Authorities. There is a summative examination integrating all foundational and basic science subjects at the end of the third year. There is also a summative examination integrating all clinical subjects at the end of the fifth year. End of posting tests are administered at the end of each internship rotation in the sixth year. Objective Structured Clinical Examination (OSCE) is administered at the conclusion of the internship year. To progress to the next phase of training, students are required to obtain a pass for each summative examination. Those who failed will have to repeat that particular stage and attempt a supplementary examination. Students are also required to attempt the Thailand Medical Licensing Examination and obtain a pass as part of graduating requirements.

## DISCUSSION

In KMU Discipline based curriculum, it allows for a systematic, comprehensive, and graduated approach to ensure a solid knowledge foundation for the medical student. This allows for more straightforward execution and control. The downside is the lack of interdisciplinary integration and collaboration between the basic sciences and clinical faculty. This result is needless duplication and encouragement of passive learning styles. There are currently ongoing efforts to effect integration of basic / foundational subjects into a systems based curriculum.

There is relatively less clinical exposure when compared to the amount of time devoted to theory sessions, resulting in substandard bedside practice and poor clinical reasoning. This results in the inability to assure the achievement of training outcomes.

Students have demonstrated a preference for interaction and discussion in the learning experience: Most of our current teaching modules employed didactic lecture methods which are teacher-centric. The result is a lack of interaction between the learner and the teacher, lack of participation by the learner in the experience, and an ignoring of the need for the learner to be active and self-directed in the process. Problem based learning and case based learning approaches are difficult to execute due to large student numbers. KMU is currently building quality web based resources to diversify and broaden the available learning tools and resources.

In MECH medical program, in context of organ systems based curriculum, the strength of this approach is that it allows for interdisciplinary integration of the learning experience. It helps students to see the big picture, and to develop a rational and structured framework that pulls together the medical, social, psychological, physiological, preventive, curative, foundational, clinical science, professionalism and scholarship domains. This approach allows for simultaneous growth in knowledge, skills and attitude. PSU deploys a diverse array of web based learning resources that affords peer to peer learning as well as faculty support for students with difficulties. Micro courses and lecture podcasts are also uploaded for student downloads and self-directed learning, achieving improvements in efficiency and quality of learning. MECH also organizes multidisciplinary learning events where faculty of the relevant disciplines is on hand to allow for a comprehensive discussion of the patient problems. This enables students to have a more holistic appreciation of the clinical problem. Teaching methods are also multi-faceted, employing lectures, outpatient clinic attachments, inpatient bedside teaching, clinical skill practice, small group learning, clinical reasoning and others. Skills towards laboratory facilities are also made available free of charge to students for practice under faculty guidance, as long as requests are made in advance.

This has a strong policy and administrative support system. Near the end of Phase 2, students are organized into groups of 15 to 20 to complete a three week community health project at a designated community health center where they are tasked to perform health education and health screens for the residents in the target community. The focus of the health education task is to teach healthy lifestyle interventions like dietary and active lifestyle promotion to reduce chronic disease incidence. The student groups are also expected to complete a project report detailing the outcomes of the interventions and submit it to the school administration. This community engagement allows students an opportunity to develop patient-doctor communication skills and build a rapport with the community.

Another limitation of the existing curriculum is a lack of coverage of commonly encountered and high prevalence conditions. Compared to the Thai model, there are also relatively fewer opportunities for case based discussion and active self-directed learning on the part of students. The differences between two education systems in China and Thailand may affect the extensibility of the research results.

## CONCLUSIONS

The focus of education changed from teachers teaching to students learning.^6^

Firstly, we should start with the integration of the medical disciplines and the humanities, so as to restore the lost “art of doctoring”.^7–8^ Secondly, we need to integrate the various foundational and clinical disciplines into an organ system based curriculum, not just in form, but also in function and purpose. Thirdly, the clinical and public health/preventive disciplines will also need to be integrated. Last but not least, the web based learning platform should be enriched with learning resources that are interdisciplinary in nature, and made easier for students to access, so as to build a culture of self-directed learning.^9^ Faculty development must be part of the solution.^10^

The focus has to be on general practice competency and practical approaches to commonly encountered conditions and problems in the community.^11^ To do this well, curriculum planners and faculty will have to be familiar with the clinical reasoning and consultation models relevant to general practice. One way will be to increase the clerkship or clinical skills practice curriculum time. Another way is to allow for easy student access to skills laboratory facilities outside of curriculum time for practice.^12^
